# List of Graduates in Rush Medical College

**Published:** 1853-03

**Authors:** 


### LIST OF GRADUATES IN RUSH MEDICAL COLLEGE,

### SESSION 1852—53.

| NAMES. | RESIDENCE. | THESIS. |
|---|---|---|
| 1. Henry Parker, - | Illinois | On the Influence of Oxygen in the Production of Disease. |
| 2. Oliver S. Jenks, - | " | On General Pathology. |

| | | | |
|---|---|---|---|
| 3. | Arthur Young, - | Illinois, | On Epidemic Cholera. |
| 4. | Warren Millar, - | ,, | On Inguinial Hernia. |
| 5. | John F. Starr, - | ,, | On the Physiology of Parturition. |
| 6. | D. Alphonn Colton, | Wisconsin | On the Effects of the Mind upon the Body. |
| 7. | Henry S. Steele, - | New York, | On Morbus Coxarius. |
| 8. | Elijah H. Drake, - | Indiana, | On Semiology. |
| 9. | J. B. Wheaton, - | Michigan, | On Etiology. |
| 10. | S. H. Whittsey, - | Illinois, | On Chemical Analysis in its Application to Pathology and Therapeutics. |
| 11. | Solon Marks, | Wis., | On Apoplexy. |
| 12. | Robt. F. Bennett, | Illinois, | On Uterine Polypi. |
| 13. | Wm. M. Young. - | Wis., | On Pneumonia. |
| 14. | James B. Moffett, | ,, | On Cholera. |
| 15. | Robt. F. Henry, - | Illinois, | On Acute Dysentery. |
| 16. | Josiah Stanley. - | Wis., | On Inflammation. |
| 17. | S. B. Harriman - | Indiana, | Acute Muco-colo-reclitis. |
| 18. | J. A. James, - | ,, | The Nutritive Functions. |
| 19. | Hosea Davis, - | Illinois, | On Remitting Fever. |
| 20. | M. F. Gerard, - | ,, | On Remitting Fever. |
| 21. | Robt. W. Earll - | Wis., | On Homæopathy. |
| 22. | Hiram Smith, - | Illinois, | On Pneumonia. |
| 23. | J. A. Brenneman, | ,, | |
| 24. | P. G. Corkins - | ,, | On Cupping in Retained Placenta. |
| 25. | A. D. Dwight - | ,, | On Puerpural Fever. |
| 26. | H. W. Ross - | Iowa, | On Malaria, its causes and effects. |
| 27. | R. Q. Wilson, - | Indiana, | On Acute Pneumonia. |
| 28. | William Curless, - | Iowa, | On Intermittent Fever. |
| 29. | Daniel Whitinger, | Indiana, | On Erysipelas. |
| 30. | James Gregory, - | ,, | On Entozoa. |
| 31. | John Philips - | Wisconsin | On Enteric Fever. |
| 32. | James M. Proctor, | Indiana, | On Symptomatology. |
| 33. | O. D. Chapman, - | Michigan, | On Puerpural Convulsions. |
| 34. | J. P. Cunningham, | Illinois. | On Typhoid Fever. |

